# Novel Human Papillomavirus Type 174 from a Cutaneous Squamous Cell Carcinoma

**DOI:** 10.1128/genomeA.00445-13

**Published:** 2013-07-05

**Authors:** Boštjan J. Kocjan, Andrej Steyer, Martin Sagadin, Lea Hošnjak, Mario Poljak

**Affiliations:** Institute of Microbiology and Immunology, Faculty of Medicine, University of Ljubljana, Ljubljana, Slovenia

## Abstract

We report the cloning and characterization of a novel human papillomavirus (HPV), now officially recognized as HPV-174, isolated from a cutaneous squamous cell carcinoma. HPV-174 has a genome organization characteristic of cutaneous HPV types, clusters to the genus *Betapapillomavirus*, species β2, and is most closely related to HPV-145.

## GENOME ANNOUNCEMENT

Human papillomaviruses (HPVs) from the genus *Betapapillomavirus* (β*-*PV) have been found in association with various benign and malignant cutaneous lesions, including nonmelanoma skin cancer in immunocompromised individuals; however, the role of these viruses in tumor formation is still unclear (1). Currently, over 40 β*-*PV types have been completely characterized and officially recognized, and are placed into 6 different species (2). We report the complete genome sequence of a novel β*-*PV type isolated from a 64-year-old male patient suffering from a facial squamous cell carcinoma.

A partial HPV L1 gene sequence of 373 bp (ENA accession number HF970576) was obtained using β*-*PV broad-range nested PCR (1) from a specimen additionally containing HPV-9 and a putative subtype of HPV-150 (FR822732). A complete viral genome was preamplified using rolling-circle amplification (3) and then amplified using inverse long-range PCR with primer pair V001FwLNG3 (5′-CCAACGAACCTTAGGCAAC-3′, nucleotides [nt] 6310 to 6328) and V001RwLNG3 (5′-TCCTGACCTGCTTTGGC-3′, nt 6309 to 6293). The resulting amplicon was cloned using a TOPO XL PCR cloning kit (Invitrogen, Carlsbad, CA) and sequenced at Microsynth AG (Balgach, Switzerland) using a primer-walking strategy. The sequence of a complete viral genome was assembled and characterized using Vector NTI Advance 11 software (Invitrogen) and phylogenetically evaluated using a maximum-likelihood algorithm, as described previously (4). A reference clone, covering the full genome of HPV-174, was deposited in March 2013 in the Reference Centre for Papillomaviruses (recently moved to Stockholm, Sweden), where its sequence was verified and the genotype officially named in May 2013.

The complete genome of HPV-174 has 7,359 bp and a G+C content of 40.2%. Its genome organization is typical of cutaneotrophic HPVs, containing a classical long control region (LCR) and five early (E1, E2, E4, E6, and E7) and two late (L1 and L2) genes but no E5 open reading frame (ORF). Multiple binding sites for transcriptional regulatory factors (such as AP-1, NF-1, and SP-1) were identified within the LCR genomic region using SIGSCAN software V4.05 (5), in addition to two consensus palindromic E2-binding sites (ACC-N_6_-GGT), two putative TATA boxes (TATAAA) of the E6 promotor, and the putative polyadenylation site (ATATAA) necessary for processing viral late gene mRNA transcripts (6, 7). Two characteristic zinc-binding domains [CxxC(x)_29-30_CxxC], separated by 36 amino acids, were identified in the putative E6 protein and one was identified in E7. The E7 protein additionally contains an LxCxE motif responsible for binding to the cell retinoblastoma protein (7, 8). The HPV-174 largest putative protein, E1, contains 601 amino acids, and the conserved ATP-binding site (GPPDTGKS) of the ATP-dependent helicase is located in its carboxyterminal part (9). The putative E4 gene has an initiation codon and is completely positioned within the E2 gene. No leucine-zipper domain was observed in the putative E2 protein (7). According to the results of phylogenetic analysis, HPV-174 clusters to the β-PV species β2 and is most closely related to HPV-145. To conclude, the genetic characterization of HPV-174 expands the heterogeneity of members of species β2 of the genus β-PV. The role of HPV-174 in the development of skin cancer remains elusive.

### Nucleotide sequence accession number.

The complete genome sequence of HPV-174 is available in the ENA, GenBank, and DDBJ databases under the accession number HF930491.

## References

[1] Potočnik M Kocjan BJ Seme K Luzar B Babič DZ Poljak M 2006 Beta-papillomaviruses in anogenital hairs plucked from healthy individuals. J. Med. Virol. 78:1673–1678 17063512 10.1002/jmv.20753

[2] Bernard HU Burk RD Chen Z van Doorslaer K Zur Hausen H de Villiers EM 2010 Classification of papillomaviruses (PVs) based on 189 PV types and proposal of taxonomic amendments. Virology 401:70–79 20206957 10.1016/j.virol.2010.02.002 PMC3400342

[3] Rector A Tachezy R Van Ranst M 2004 A sequence-independent strategy for detection and cloning of circular DNA virus genomes by using multiply primed rolling-circle amplification. J. Virol. 78:4993–4998 15113879 10.1128/JVI.78.10.4993-4998.2004 PMC400362

[4] Kocjan BJ Hošnjak L Seme K Poljak M 2013 Complete genome sequence of a novel human betapapillomavirus, HPV-159. Genome Announc. 1(3):e00298-13. 10.1128/genomeA.00298-13 23723399 PMC3668007

[5] Prestridge DS 1991 Signal scan: a computer program that scans DNA sequences for eukaryotic transcriptional elements. Comput. Appl. Biosci. 7:203–206 2059845 10.1093/bioinformatics/7.2.203

[6] Tachezy R Duson G Rector A Jenson AB Sundberg JP Van Ranst M 2002 Cloning and genomic characterization of Felis domesticus papillomavirus type 1. Virology 301:313–321 12359433 10.1006/viro.2002.1566

[7] de Villiers EM Gunst K 2009 Characterization of seven novel human papillomavirus types isolated from cutaneous tissue, but also present in mucosal lesions. J. Gen. Virol. 90:1999–2004 19386784 10.1099/vir.0.011478-0

[8] Lehoux M D’Abramo CM Archambault J 2009 Molecular mechanisms of human papillomavirus-induced carcinogenesis. Public Health Genomics 12:268–280 19684440 10.1159/000214918 PMC4654617

[9] Titolo S Pelletier A Sauvé F Brault K Wardrop E White PW Amin A Cordingley MG Archambault J 1999 Role of the ATP-binding domain of the human papillomavirus type 11 E1 helicase in E2-dependent binding to the origin. J. Virol. 73:5282–5293 10364274 10.1128/jvi.73.7.5282-5293.1999 PMC112583

